# Simulation of nano elastic polymer chain displacement under pressure gradient/electroosmotic flow with the target of less dispersion of transition

**DOI:** 10.1038/s41598-021-99093-3

**Published:** 2021-10-04

**Authors:** Ramin Zakeri, Eon Soo Lee

**Affiliations:** 1 Department of Mechanical Engineering, Shahrood University of Technology, Shahrood, Iran; 2 Department of Mechanical & Industrial Engineering, NJIT, Newark, NJ USA

**Keywords:** Biomedical engineering, Mechanical engineering

## Abstract

Since non-scattering transfer of polymer chain in nanochannel is one of the important issue in biology, in this research, the behavior study of a long polymer chain in the nanofluid in two modes of free motion and restricted motion (fixed two ends) under two different forces including constant force (pressure gradient (PG)) and variable force (electroosmotic force (EOF)) has been investigated using dissipative particle dynamics (DPD) method. Our aim is that displacement of polymer chain carries out with less dispersion. Initially, without the presence of polymer, the results have been validated in a nanochannel by analytical results for both cases (PG, EOF) with an error of less than 10%. Then, assuming 50 beads of polymer chain, the polymer chain motion in free motion and fixed two ends modes has been examined by different spring coefficients between beads and different forces including PG (0.01 DPD unite) and EOF (zeta potential =  − 25 mV, electric field = 250 V/mm, kh parameter = 8). The results show that in free polymer motion-PG mode, by increasing 1.6 times of spring coefficient of the polymer, a 40% reduction in transition of polymer is achieved, which high dispersion of polymer chain is resulted for this mode. In the EOF, the spring coefficient has a slight effect on transferring of polymer and also, EOF moves the polymer chain with extremely low polymer chain scattering. Also, for fixed two ends-PG mode, a 36% reduction in displacement is achieved and in the same way, in EOF almost 39% declining in displacement is resulted by enhancing the spring coefficients. The results have developed to 25 and 100 beads which less dispersion of polymer chain transfer for free polymer chain-EOF is reported again for both circumstances and for restricted polymer chain state in two PG and EOF modes, less differences are reported for two cases. The results show that the EOF has the benefit of low dispersion for free polymer chain transfer, also, almost equal displacement for restricted polymer chain mode is observed for both cases.

## Introduction

From biology and medicine to space exploration, the subject of micro/nanofluid transfer along with solid nanoparticles or polymer nanofibers has been always important^1–3^. For example, DNA analysis devices use a microfluidic system for DNA transfer and analyzing and separations. Also, transfer of nanoparticles in fluid flow is an efficient method for cooling. All these applications have two common challenges, a method for pumping the fluid flow and collision effect of fluid particles on nanoparticles or polymers or DNA transfer^3–9^.

For the first challenge, several fluid transfer methods have been presented, such as electrosmotic micropumps^10^ (Interaction of Electric field motion and electrostatic surface), magnetic hydrodynamics^11^ (magnetic field motion and Lorentz force), piezoelectric^12^ (electric field vibrations), acoustic^13^ (pressure wave propagation) and also, one of the common practical and simple methods of particle fluid transfer is micro-pressure gradient pump. In the pressure gradient method, by creating a constant pressure gradient, all fluid particles begin to move under the influence of a constant force^14^.

Considering second issue, the collision of fluid particle with nanoparticles or polymers and how they transfer is a significant challenge. In this regard, the transfer of polymer by microfluid and micropump has been studied by researchers to investigate various applications such as polymer chain transfer, DNA, separation and different species and blood cells^15–17^. One of the most important issues in transmission is the non-scattering of polymer or DNA chains with other materials, and transmission without scattering is very important^15–17^. One case in point is the study of the behavior of polymer chains or their complex behavior in nano/microfluids. The study and analysis of polymer chain movement in various fields of study such as biology, genetics, etc. are very essential. Transfer of polymers by micro/nanofluid is observed in many biological processes in living cells or chemical processes through narrow pores, protein transfer through cell membranes and the penetration of viruses into the cell nucleus. Knowledge of the dynamics behavior of the polymer chain is very useful in the design and fabrication of transmitter microsystems as well as the behavior of the biosystem or drug delivery^18–20^.

Due to mentioned challenge, one of the important method for fluid transfer is electroosmotic flow which although is a proper method for transition of nanoparticles or polymer chain in a biofluid but there are several unknown phenomena in this type of fluid transfer. Various researchers have simulated EOF phenomena in the macro scale using the computational fluid dynamics (CFD) simulation method^21^. Also, in order to study the flow simulation in more detail, researchers used non-continuous fluid methods to simulate these phenomena at the nanoscale in a simple channel^22–26^. The study of the effective parameters of electroosmotic flow is also one of the important topics that has been done in order to better understand this phenomenon^27^. Using the proper simulation method, for investigation of electroosmotic flow in nano scale, plays important role for finding details of movement of fluids and consequently transfer of a polymer chain through fluid flow^28^.

As second challenge mentioned, complex behavior from collision of particles are formed and will affect on polymer motion in nano channel. Numerical simulation is a promising tool for understanding the complex behavior of polymer chains^29^. Clearly, the particle methods for simulation are able to show more details. The most important and classic method of simulation is the molecular dynamics method. This method is used in very small nanometer scale and is not suitable for simulation of many application microsystems because it has very high computational costs, although simulation with this method is very close to real physics. To overcome this limitation, researchers have sought some methods that are less computationally costly but at the same time closer to the actual physics of non-continuous and molecular fluids^30^. The latest method proposed is the dissipative particle dynamics (DPD) method. The DPD method is a mesoscopic method that it has been widely used in micro/nanoscale simulations recently. In this method, using molecular clusters, known as particles, instead of considering all real molecules, all the motion and collision of clusters or particles are considered as a group and has a lower computational cost compared to the molecular dynamics method^31,32^.

As a result, DPD method can solve two challenges, thus various researchers have been using this method for simulation of motion of nanoparticles or polymers through fluid flow using pressure driven or electroosmotic external force or other external forces. Manke and Zhang^33^ used the DPD method to study the dynamics and rheology of polymer solutions and suspensions consisting of spherical particles with adsorbed polymers. They used constant external force and they found that both polymer solutions and polymer domain suspensions have Newtonian behavior with low shear rates, while they perform thin shear behavior for higher shear rates. Wilmson et al.^34^ used the DPD method to study the behavior of a polymer within a capillary and the effect of polymers on the melting process in a shear flow considering constant external force. Based on the DPD method, Duong Hong et al.^35^ proposed an electrophoresis model for DNA that was able to simulate the electroosmotic and electrophoretic (variable forces) motion of the accompanying DNA in the micro/nanoscale channel. Using this model, they were able to obtain free-moving DNA discharge while avoiding expensive electrostatic interactions in molecular simulations. They also calculated the movements of the particles in realistic geometry with great precision. Pan et al.^36^ used the DPD method (constant external force) to study the separation of diets in a micro device using the entropic trapping mechanism. They showed that longer strands of DNA were faster than shorter strands. They concluded that entropic trapping was the result of delayed entry. In addition, they concluded that trapping the particles in a corner did not help to isolate the DNA. Guo et al.^37^ studied the effect of elastic chains in nanochannel using the DPD method with assumption of constant force. Ranjiz et al.^38^ studied single and bilayer layers of polymer coatings using DPD method (constant external force). They focused on possible differences between the link ring and the linear polymer chains in relation to the radius of rotation, roughness, orientation, and density profiles as functions of distance from the link plate. They also studied the laws of the gyration radius and normal and parallel components. Zakeri^39^ used the DPD method to simulate the performance of a soft polymer micro-stimulator in an electrosmotic flow (variable force) in a simple micro-channel and a divergent convergent.

Since the control motion of polymer chain or DNA is essential^40,41^, considering the pressure gradient (constant external force) and electroosmotic flow (variable external force), the movement of the polymer chain or displacement of polymer chain in a period of time from initial to final position in free mode (free polymer chain) and restricted conditions (fixed two ends of a polymer chain) for finding more about complex behavior of a polymer chain in nanoflow and improvement of non-dispersion of transferring of polymer chain have been compared and investigated using DPD method.

## Numerical simulation and the present model

As mentioned, the molecular dynamics method considers each particle as a molecule and the collisions between particles are according to Newton’s second law. The temporal and longitudinal scales of this method are very small and if we want to use it in larger scale (mesoscale), it will have a high computational cost. In the particle dynamics method, the number of molecules are considered as a particle. The mesoscopic method is a coarse-grained method in which each particle with mass of ${m}_{i}$ represents a large number of molecules. The relationship between velocity (${\overrightarrow{v}}_{i})$, position (${\overrightarrow{r}}_{i})$, and force between particles, like the molecular dynamics method, is written according to the relationship of Newton’s second law, which is presented below^42,43^:

1 $${\overrightarrow{v}}_{i}=\frac{d{\overrightarrow{r}}_{i}}{dt},$$

2 $${m}_{i}\frac{d{\overrightarrow{v}}_{i}}{dt}={\overrightarrow{F}}_{i}.$$

In general, the force ${\overrightarrow{F}}_{i}$ consists of two general external and internal forces. The external force is the same as the magnetic or electroosmotic force, and the internal force is the intermolecular force, which includes the three survival forces, the random ${\overrightarrow{f}}_{ij}^{\mathrm{R}}$ and the dissipation ${\overrightarrow{f}}_{ij}^{\mathrm{D}}$, which are listed below^42,43^:

3 $${\overrightarrow{F}}_{i}=\sum_{j\ne i}{\overrightarrow{f}}_{ij}+{\overrightarrow{F}}_{ext},$$

4 $${\overrightarrow{f}}_{ij}={\overrightarrow{f}}_{ij}^{\mathrm{C}}+{\overrightarrow{f}}_{ij}^{\mathrm{D}}+{\overrightarrow{f}}_{ij}^{\mathrm{R}},$$

note that the two forces of dissipation and random are entered into the calculations because this method, unlike the method of molecular dynamics, examines the collision of the representatives of molecules and the creation of two forces of dissipation and random based on the mathematics of cluster collision is mandatory. It should also be noted that the external force in this research would be pressure gradient (PG, constant force) and electroosmotic flow (EOF, variable force). This term is the same as the source term in the CFD which is used to account for external force. Conservative force is derived from the internal force classification of Leonard Jones mediation, which is defined as follows^42,43^:

5 $${\overrightarrow{f}}_{ij}^{\mathrm{C}}=\left\{\begin{array}{l}{a}_{ij}\left(1-{r}_{ij}/{r}_{\mathrm{c}}\right){\widehat{r}}_{ij} {r}_{ij}<{r}_{\mathrm{c}}\\ 0 {r}_{ij}\ge {r}_{\mathrm{c}}\end{array}\right..$$

In the above relation ${a}_{ij}$ is the collision coefficient of two particles i and j and outside the collision radius is considered zero. Based on the DPD formulation, ${r}_{ij}=\left|{\overrightarrow{r}}_{ij}\right|$, ${\widehat{r}}_{ij}={\overrightarrow{r}}_{ij}/\left|{\overrightarrow{r}}_{ij}\right|$ and ${a}_{ij} = 75{k}_{b}T/\rho$. Also, the other two terms of the internal forces of dissipative force and random force are calculated by relations (10), respectively:

6 $${\mathbf{F}}_{ij}^{D}=-\gamma {\omega }^{D}\left({r}_{ij}\right)\left({\widehat{\mathbf{r}}}_{ij}\cdot {\mathbf{v}}_{ij}\right)\left({\widehat{\mathbf{r}}}_{ij}\right),$$

7 $${\mathbf{F}}_{ij}^{R}=\sigma {\omega }^{R}({r}_{ij}){\theta }_{ij}{\widehat{\mathbf{r}}}_{ij.}$$

${\theta }_{ij}$ is a random function with zero mean properties and a single variance. In the above equations $\sigma$ and $\gamma$, respectively, the power factor for acceleration and random forces ${\mathbf{v}}_{ij}$ = (${\mathbf{v}}_{i}$− ${\mathbf{v}}_{j}$), and ${\omega }^{D}$ and ${\omega }^{R}$ are two weight functions, which are calculated in the following equation:

8 $${\omega }^{D}\left({r}_{ij}\right)={\left[{\omega }^{R}\left({r}_{ij}\right)\right]}^{2}=\left\{\begin{array}{l}\sqrt{1-\frac{{r}_{ij}}{{r}_{c}}}, {r}_{ij}<{r}_{c}\\ 0, {r}_{ij}\ge {r}_{c}\end{array}\right..$$

As mentioned, the polymer chain consists of a number of beads and springs between them. This internal force should be added to ${\overrightarrow{f}}_{ij}^{\mathrm{C}}$. To simulate the presence of a polymer chain in the flow, the force calculation field includes particle-to-particle collision, particle to polymer beads collision, and polymer bead to polymer bead collision, which must be taken into account in the calculation of internal force. In other words, particle collisions include bead-to-bead collisions with bead to fluid particles. Also, between the items in this type of spring simulation, it is assumed that it is necessary to calculate the spring force and add it to the survival force. Due to the collision of fluid particles or spherical particles with each other, they cause the spherical particles to move relative to each other and the spring force is increased^34,43–45^:

9 $${\mathbf{F}}_{ij}^{p}=-K{\left({r}_{ij}\right)}_{p}{r}_{ij}.$$

**Figure 1:**
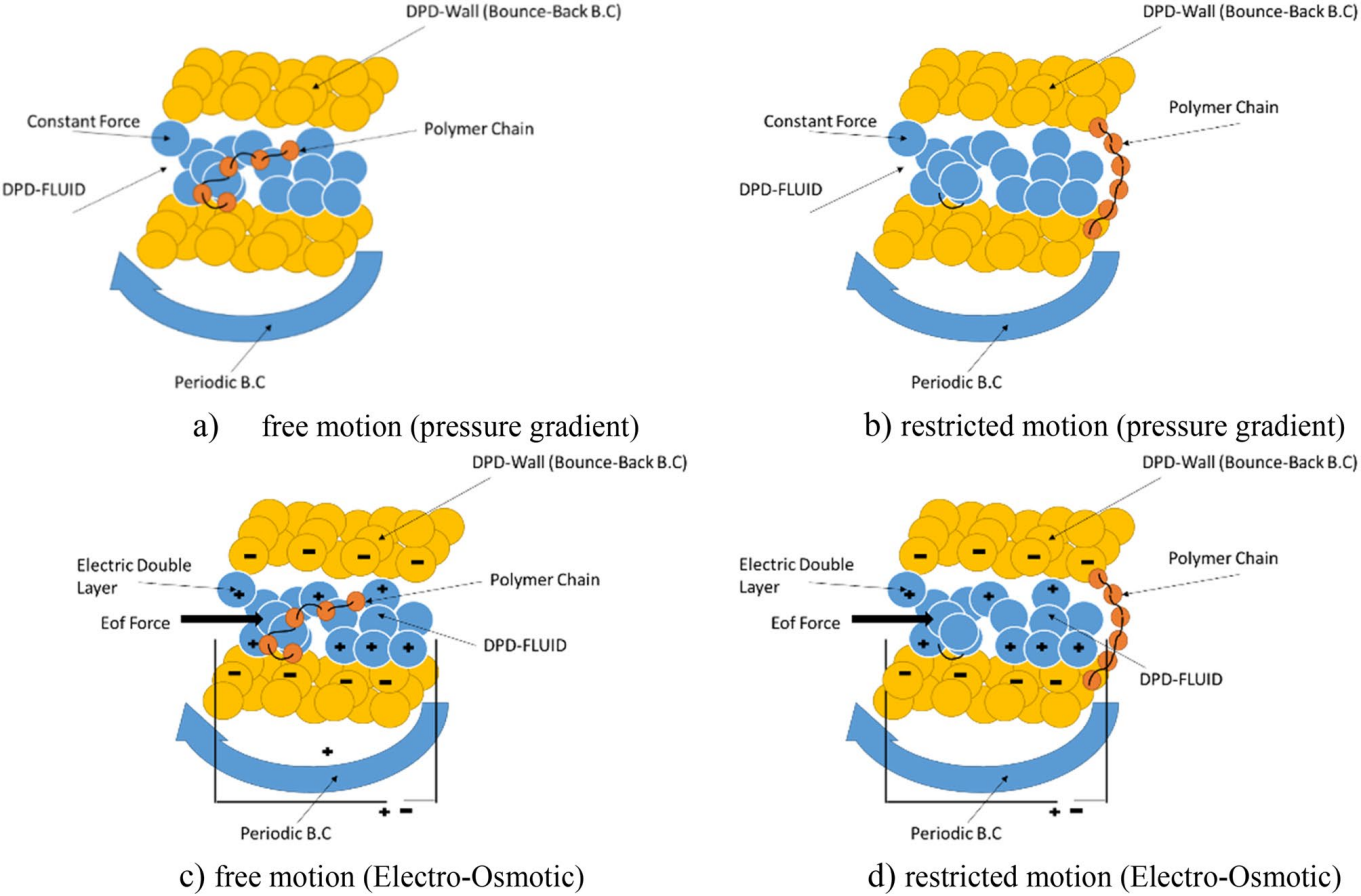
Schematic of DPD particle motion in a channel with periodic and bounce-back boundary condition and presence of polymer chain in nanochannel in two cases of free motion (**a,c**) and restricted motion of polymer chain (**b,d**) for pressure gradient and electroosmotic force.

In the above relation $k{\left({r}_{ij}\right)}_{p}$ is the spring coefficient which is considered the same for all items in the present simulation. In the force field calculation loop, the spring forces between the beads are calculated and added to the conservative force at the moment the DPD grains hit the beads or bead to bead, and the force field is updated^36^. The model in this research is a polymer chain in the nanochannel that moves under the constant force of nanofluid particles and causes a change in the state of the polymer chain, which is like connecting a number of beads and springs to each other. The walls of the channel are made of solid particles and are returned to the field of motion when the fluid particles collide. Determination of fluid material, walls and polymer chain beads was explained numerically. As the particles leave the channel, a periodic boundary condition causes the particles to return to the computational field at the beginning of the channel. It should be regarded that based on the DPD formulation^20,42^, overlapping of particles is the main characteristic of soft matter. Also, amount of behavior of particles depends on several parameter such as ${a}_{ij}$ and external forces. Figure 1 shows the introduced model, which in the next section, the results of polymer movement in the channel will be reviewed and analyzed.

The external force in this simulation includes the electro-osmotic force as well as the constant pressure gradient force. In the case of electroosmotic force, it includes two important terms net charge density ($${\rho }_{e})$$ and electric field ($${E}_{0})$$:

10 $${\overrightarrow{F}}_{ext}={E}_{0}{\rho }_{e},$$

the net charge density can be achieved by Poisson-Boltzmann equation:

11 $${\nabla }^{2}\Psi =-\frac{{\rho }_{e}}{\upepsilon },$$

where $\Psi$ is electric potential and $\upepsilon$ is the relative permittivity of the solvent in free space permittivity. Based on the EOF equations, the effective parameters include zeta potential (depend on material of wall), electric field and effect of ionic concentration or dimensionless parameter of kh parameter (height of channel $\times$ inversed electric double layer thickness)^22–26^.

The Verlet algorithm is used to solve the above equations. According to this algorithm, with the initial assumption of velocity values, the displacement is initially calculated and then the forces between particles are calculated and in the iteration loop a new velocity and a new position are calculated and the forces field, the velocity and position values of the particles are updated again.

To develop our result for finding more about polymer chain behavior in free and restricted modes, the radius of gyration and the velocity auto-correlation function (VACF) would be so effective. The radius of gyration formulation is

12 $${R}_{G}^{2}=\frac{1}{n}\sum_{1}^{n}{\left({r}_{i}-{r}_{mean}\right)}^{2}=\frac{1}{{2n}^{2}}\sum_{ij}{r}_{ij}^{2},$$

in which, *n* is the bead number of a polymer, ${r}_{mean}$ is the mean position of the polymer chain and ${r}_{ij}=\left|{\overrightarrow{r}}_{i}-{\overrightarrow{r}}_{j}\right|$. Also, formulation of VACF is, at the first, should be calculated^23,45^:

13 $$ V_{{vacf}} \left( {it} \right) = \sum\limits_{{i = 1}}^{{it}} {v_{p} \left( {it_{{t0}} } \right)} v_{p} \left( {it_{{t + t0}} } \right), $$

where ${v}_{p}$ is the velocity of the center-of-mass of the polymer chain $,$ it is the iteration number of time step, $\left({it}_{t0}\right)$ and $\left(i{t}_{t+t0}\right)$ are the initial time of polymer motion and summation of initial time with delay time.

From analytical aspect of EOF, using the momentum equation for simple channel, the analytical solution can be derived (see Eq. (10))^10,20^:

14 $$\frac{d}{dy}\left(\mu \frac{d{v}_{x}}{dy}\right)-{E}_{0}{k}^{2}\varepsilon\Psi =0,$$

considering linearized Poisson-Boltzmann equation for simple channel as follows:

15 $$\frac{{d}^{2}\Psi }{d{y}^{2}}={k}^{2}\Psi ,$$

where $k$ is the inverse of Debye length, *h* is one-half of the channel height and $\mu$ is the viscosity of fluid, the boundary conditions can be described as

16 $$\Psi \left(\mathrm{at y}=\mathrm{h}\right)=\mathrm{\xi and }\frac{d\Psi }{dy} \left(at y=0\right)=0,$$

where $\upxi$ is the zeta potential. Solving Eq. (15) using the two boundary conditions (Eq. (16)), it eventually can be written as

17 $$\Psi \left(\mathrm{y}\right)=\upxi \frac{\mathrm{cosh}(ky)}{\mathrm{cosh}(kh)},$$

considering Eq. (14), two boundary conditions for a simple channel should be applied as

18 $${v}_{x}\left(at \, y=h\right)=0 \,{\text{and}}\, \frac{d{v}_{x}}{dy}(at\, y=0)=0,$$

thus, analytical solution can be presented as

19 $${v}_{x}\left(y\right)=\left(\frac{-\varepsilon {\upxi E}_{0}}{\mu }\right)\left(\frac{{\int }_{ky}^{kh}\mathrm{sinh}\left(ky\right)d\left(ky\right)}{\mathrm{cos}\left(kh\right)}\right).$$

## Results

In this section, investigations have been followed to study the complex behavior of a polymer chain in two modes of free movement and fixed two ends of a polymer under different external forces. Our aim is that to find which mode enjoys advantages of less polymer beads dispersion under driven forces. The driven forces for this DPD simulation are under the influence of constant force (PG or pressure gradient) and variable force (electroosmotic force or EOF). First, the results for a simple channel are compared with the analytical results, and then the above cases are investigated to study the complex behavior of the polymer chain in different states. Since non-dispersion of transferring of polymer chain is an important issue in biological science such as DNA transfer, in this study, this point has been considered in this regard.

### Validation of DPD simulation in nano channel

As mentioned, the dissipative particle dynamics is a reliable method for simulating non-continuous fluids in a multi-nanometer-to-micro scales. This method has the advantage of having a larger time and length scale compared to molecular dynamics method and the same as the lattice Boltzmann method^11^ (reducing the computational cost) while by increasing the number of degrees of freedom (advantage over the lattice Boltzmann method) will give more realistic simulation than the LBM or CFD method. The geometry in this study consists of a simple channel in which DPD particles are placed and the walls are made of particles and the bounce back condition is applied. The particles are also directed to the beginning of the channel by implementation of periodic boundary condition when they reach the end of the channel. The particles are driven by constant and variable external forces that causes the particles to be pushed forward.

**Figure 2:**
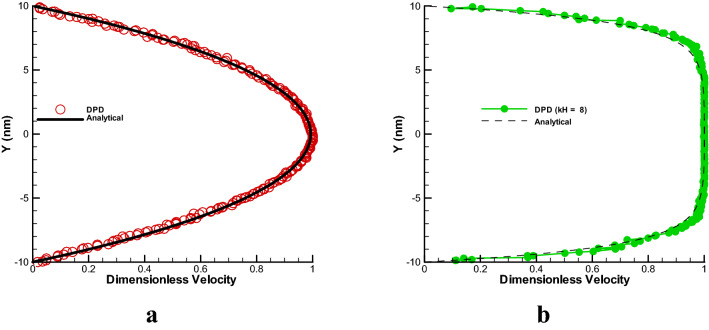
Comparison of DPD (circle) and analytical (continuous line) simulation solution of velocity profiles in simple channel for constant force of 0.01 ((**a**), pressure gradient (PG)) and variable force ((**b**) electroosmotic force (EOF); zeta potential − 25 mV, electric field 250 V/mm and kh = 8).

The simulation condition in a simple channel under the influence of the constant force of the pressure gradient and electroosmotic force is given in Table 1. In Fig. 2, the DPD method is used for simulation of both cases and the velocity profile diagram are compared by analytical solution^10,20^. As the particles move by a constant force (2-a), the particles that are close to the wall are more affected by the shear force and have a lower velocity, while as the distance from the wall increases, the shear force decreases and the velocity of the particles increases. Note that the non-continuous motion of particles and the collision of particles always fluctuate in the actual physics of flow. The accuracy of the results show that less than 10% of the difference is reported between the results and the analytical results^20^. In the case of electroosmotic force (2-b), the electric double layer provides motion which the whole fluid will move and for mentioned condition, a plug shape velocity profile will form finally in fully develop state. The effecting parameters involves zeta potential, electric field and kh parameter which the study of these parameter had been done in Refs.^23–27^. The accuracy of this method is less than 10% compared to analytical results^10^. As mentioned, this percentage of difference is due to such fluctuations in particle motion that it is inherent in the Lagrangian solution method and is closer to the real physics of particle motion.

**Table 1:** Constant values for simulation of nanofluid/polymer motion in the nanochannel.

Parameter	Value
Number of particles	4000
Dimension of channel (nm)	20 $\times$ 20 (nm)
Boundary condition	Bounce—back (wall) and periodic^31^
Repulsion parameters fluid–fluid and fluid-wall, fluid-polymer, polymer–polymer^20,42^	25, 8, 25, 8
**PG (constant force) parameters**	
Pressure gradient constant	0.01
Time step for PG (s)	10^−2^
Number of time step for PG	1000
**EOF (variable force) parameters**	
Electric field	250 V/m
Zeta potential	− 25 mV
kh parameter	8
Time step for EOF (s)	10^−3^
Number of time step for EOF	10,000

### Free polymer chain motion under EOF and PG

In this section, a polymer chain is placed in the channel (Table 1) away from the channel walls and the movement of the polymer is investigated under the influence of a constant (pressure gradient) and variable (electroosmotic) external force. As mentioned in previous section, velocity profiles of PG and EOF, because of EOF and PG forces distribution, are different and these variations will affect on collision of particles and consequently transfer of polymer chain. As can be expected, when particles collide the beads, considering the spring stiffness coefficient, it will causes the momentum transfer to polymer chain and consequently, motion of polymer chain will be observed in the nanochannel but pressure gradient or electroosmotic flow will have different pattern in transfer of a polymer chain which more investigation is necessary to find less dispersion of displacement.

It is noteworthy that the materials such as wall material, fluid material and polymer material are applied by considering the repulsion coefficients (Eq. (5)) in the DPD method. Also, considering the height of wall 20 nm, placing a polymer chain almost in the middle of the channel causes the effect of the wall to be greatly reduced, and also putting polymer chain anywhere approximately in the middle has no effect on the amount of displacement. Also, Note that two types of boundary conditions are used in this simulation, namely the bounce back boundary condition used in the channel wall and the periodic boundary condition in which particles are directed to the beginning of the channel when they reach the end of the channel. The polymer is brought to the beginning of the channel by reaching the end of the channel. It should be mentioned that the initial position of polymer chain in channel is vertical form, and displacement, based on our target, should be carried out with at least dispersion.

**Figure 3:**
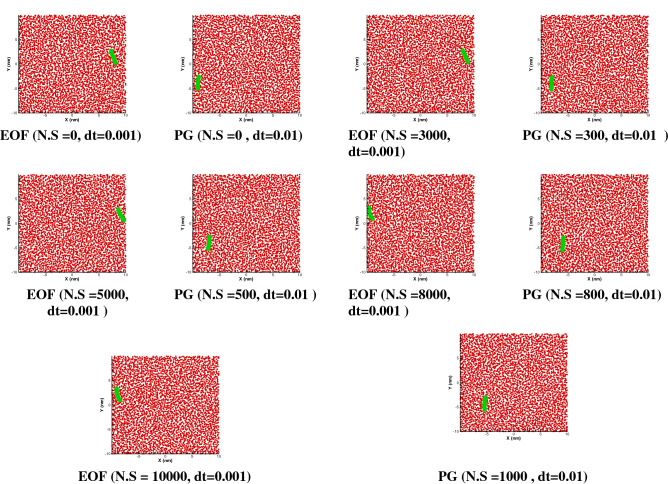
Free movement of the polymer chain in a simple channel under the influence of a constant external force (PG of 0.01 DPD unite) and electroosmotic force (EOF, Table 1) with spring coefficient of 8000 between the beads over number of steps (N.S) and time step for n = 50.

Figure 3 shows the comparison of motion of a polymer chain (spring coefficient of 8000) with pressure gradient and electroosmotic force (Table 1). How the polymer moves depends on collision between particles and beads, the type of external force and springs stiffness between beads. Based on Eq. (5), we have several types of collisions, including the collision of fluid particles with each other, fluid to polymer, and polymer to polymer. The polymer is also affected by this collisions field and shows a complex motion and transfer. The question here is what the effect of changing the spring coefficient (polymer type) and types of external forces would be on the displacement rate and dispersion of polymer particles, which is investigated as follows.

Due to external force effect, the DPD particles move forward and consequently, the polymer will change its position. There is no restriction to prevent the polymer chain from moving over time. Based on the results of Fig. 3, the electroosmotic force provides a smoother motion compared to pressure gradient. Considering the nature of this type of flow, it is clear that a gradual motion starts from the electric double layer and then spreads to the rest of fluid flow, while in the pressure gradient, a constant force is applied to all particles and all particles will collide together with higher intensity. Results show that for K = 8000 both cases have low dispersion in transfer of polymer chain while a pressure gradient provides higher displacement.

**Figure 4:**
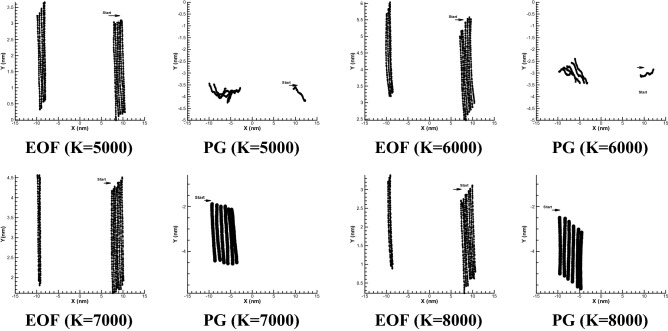
Free movement of the polymer chain in a simple channel under the influence of a constant external force (PG of 0.01 DPD unite) and electroosmotic force (EOF) with different spring coefficient between the beads over time for n = 50.

To further investigate the issue, the stiffness ratio between the beads has been changed to affect on how it moves. Figure 4 shows the different modes of motion of the polymer, considering the spring coefficients of 5000, 6000, 7000 and 8000, under constant and variable external force. As can be seen, electroosmotic flow provides more uniform polymer chain distribution which the oscillation of polymer chain will be less compared to pressure gradient force. So to speak, pressure driven gradient has higher dispersion especially in the case of low spring coefficient. Also, in both cases, with increasing the spring coefficient of the polymer, not only does the polymer has less displacement but also it tends to keep its position and stand vertically. For pressure gradient test case (PG), by increasing the spring coefficient (6000 or more for example), the polymer tends to keep standing vertical or initial position while by decreasing the spring coefficient (5000 or less for example), the polymer losses its initial position and it becomes more horizontal state which it will form with more stretch or even compression (depend on flow) in more horizontal direction. This trend can be followed in mentioned Fig. 4.

Overall, for pressure gradient test case (PG), with a 1.6 time increase in spring coefficient (from 5000 to 8000), a 40% reduction in polymer displacement is observed while for elctroosmotic flow, the displacement significantly has not been affected by spring coefficient. It is concluded that EOF provides significant less dispersion for full range of stiffness coefficient compared to pressure driven flow. Thus, this type of polymer transition, without mixing or any scattering of polymer beads, would be more proper than pressure driven case.

**Figure 5:**
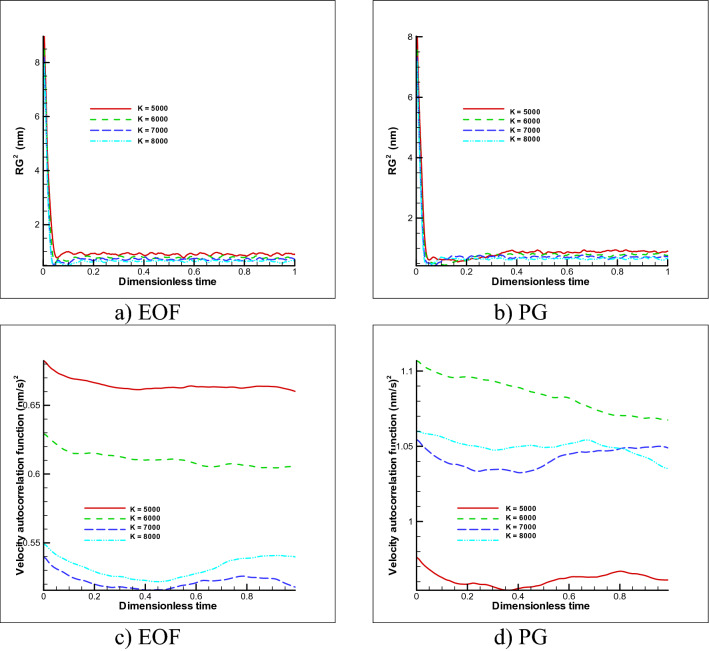
Changes in gyration radius (**a,b**) and velocity correlation function (**c,d**) over time for free movement of the polymer chain in different spring coefficient and different external forces (n = 50).

Then, to investigate the behavior of the polymer chain over time steps, the two parameters of gyration radius and velocity correlation function are investigated in Fig. 5 for both cases. The first parameter shows the size of random coil shape of the polymer chain over time and the second parameter shows the dynamics of the polymer chain over time. As expected, as the stiffness coefficient increases, the gyration radius also decreases because the size of springs decreases. It should mention that at beginning numerical run, due to effect of initial condition, amount of gyration radius should be ignore (non equilibrium condition) and after short time, the correct numerical value is reported. Also, the velocity correlation function shows velocity fluctuations in different stiffness coefficients that has not a specific decreasing or increasing trend and only it shows that velocity turbulence in all cases will decrease over time. The above study shows that with increasing the spring coefficient of the polymer, it tends to have less dispersion and less movement occur for both cases. For electroosmotic case in this regard, both study parameters show the same behavior of pressure driven flow with less reported values. Thus, the electroosmotic flow provides less dispersion in transfer of a free polymer chain as reported in previous results too.

### Restricted polymer chain motion under EOF and PG

Despite the free movement of polymer chain in nano channel, restricted movement of polymer chain is not able to displace easily. By adding this condition which it has several applications such as nano sensors or nano actuators, it would be essential to investigate what is the effect of PG and EOF on displacement of restricted polymer chain and amount of dispersion.

Next, in order to develop the results, it is considered the fixed two ends of a polymer chain (first and last beads of polymer chain). Due to the movement of DPD particles by constant or variable force, the interaction of fluid particles with polymer will cause to transfer the momentum and the polymer chain tends to stretch. It should be considered that because of tension/compression of spring between beads and fixed two ends of a polymer, a restricted motion of polymer is formed over time steps.

**Figure 6:**
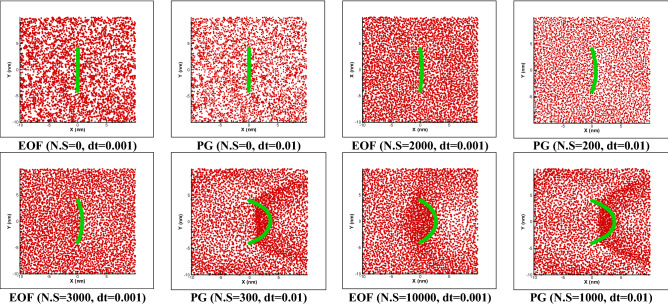
Restricted movement of the polymer chain in a simple channel under the influence of a constant external force (PG of 0.01 DPD unite) and electroosmotic force (EOF, Table 1) with spring coefficient of 8000 between the beads over number of steps (N.S) and time step for n = 50.

In Fig. 6, fixed two ends of a polymer is installed at points 4 and − 4, in which the tendency to stretch appears under the influence of the PG or EOF. By passing time, the amount of stretching increases to certain point, this is because collision of particles together and momentum transfer from one side, and strength of spring from another side will reach a balance between forces. Based on the reported result, for a polymer contains 50 beads, a spring coefficient of 8000 and a constant force of 0.01 almost 20% displacement is resulted in relative to the channel width for PG and 17.5% for EOF is reported which slight lower displacement is observed compared to pressure gradient. Two points should be mentioned: first, for both PG and EOF cases by increasing the spring stiffness, amount of displacement decreases linearly and second, polymer displacement for EOF test case is lower than PG.

**Figure 7:**
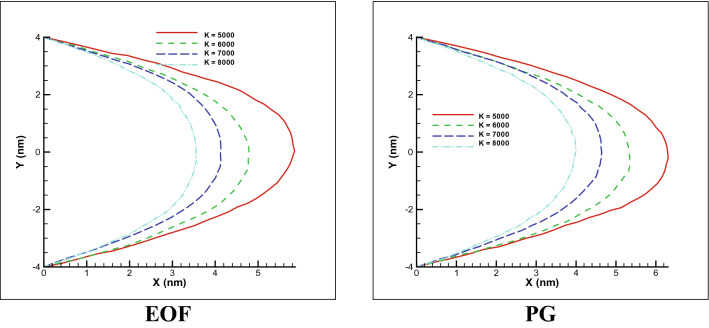
Movement with fixed two ends of a polymer chain in a simple channel considering different stiffness coefficient for both pressure gradient and electroosmotic flow test cases (n = 50).

One of the things that we are looking for in this research is the effect of changing the spring stiffness and effect of different external forces on the displacement rate of the polymer chain. By changing the spring coefficient in the pressure gradient test case, the total amount of polymer stretch would be higher to electrosmotic flow and is shown in Fig. 7. Also, both cases, amount of displacement will be decreased by enhancing the spring coefficient. In this study, by increasing the spring coefficient by 1.6 times (from 5000 to 8000), the displacement of polymer chain decreases almost 36% and for electroosmotic flow, almost 39% decreasing in stretching is observed in this study.

From obtained results, total displacement of electroosmotic a minor amount is lower compared to pressure gradient test case. This is because the nature of EOF is more uniform (plug like velocity profile). This result states that for more uniform transfer of restricted polymer chain, EOF is a proper choice especially important point is that we can control the fluid flow using changing the electric field which this method will have several applications such as nano actuators. It should be notice that control of PG is not easy as much as EOF. The displacement process is not completely linear due to the spring effect, but the restricted displacement process is more linear than the free polymer displacement mode, which is further explained at the end of the section.

**Figure 8:**
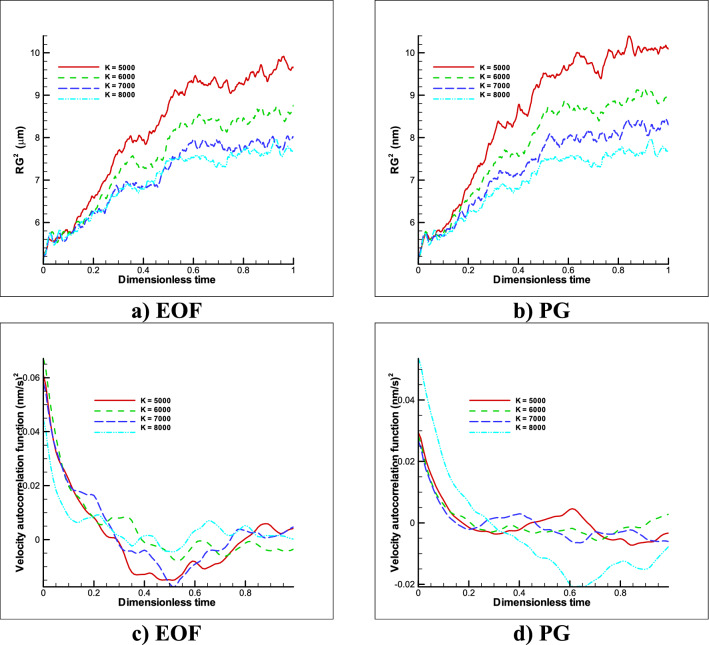
Changes in gyration radius (**a,b**) and velocity correlation function (**c,d**) over time for restricted movement of the polymer chain in different spring stiffness (bond coefficient) states and different external forces (n = 50).

In order to investigate the complex behavior of the polymer over time, two properties of the polymer chain motion have been studied, which are the gyration radius and the velocity autocorrelation function. By reducing the spring coefficient, the polymer stretch more easily, and radius of gyration will clearly increase. Also, the correlation function shows, as mentioned before, the velocity turbulence in motion, but it does not show a specific decreasing or increasing trend such as the radius, of gyration which is shown in Fig. 8. This study, as in the previous case, shows that with increasing the spring coefficient, the polymer displacement decreases and the tendency to maintain the initial state is higher.

**Figure 9:**
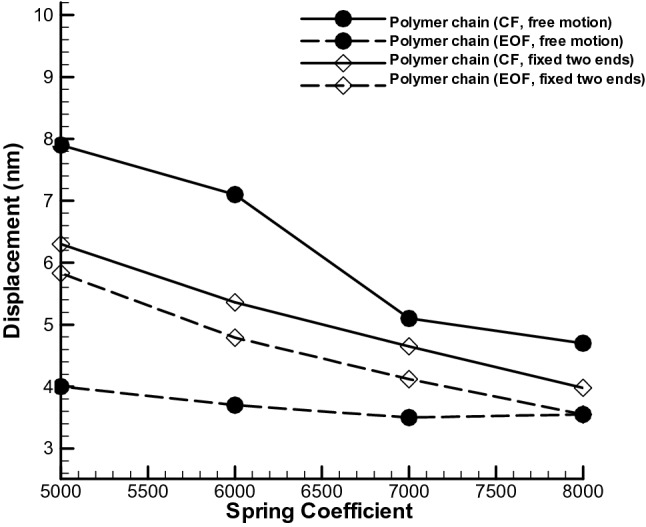
Polymer chain displacement to spring stiffness coefficient between beads for two modes of free and restricted movement for pressure gradient and electroosmotic test cases (n = 50).

Figure 9 shows a comparison diagram of the displacement of the polymer chain for specific time range, considering different spring constants and external forces (PG and EOF), in the two modes of free movement and motion with restricted mode. In the case of pressure gradient, as can be seen for both free and restricted polymer chain, a decreasing behavior in displacement is observed by increasing spring stiffness.

In the free motion mode, with increasing spring coefficient, amount of displacement decreases and size of polymer chain and coil form of polymer chain reduce for PG test case while for EOF test case, polymer chain shows a small variation for all spring coefficient ranges. Also, in restricted motion mode, both test cases show a linear declining form by enhancing the spring coefficients. This is because, by moving the particles and colliding with the fixed two ends of a polymer, the momentum is transferred more efficiently, and some particles are trapped in the chain, and the momentum transfer is performed better, and Fig. 9 shows higher linear displacement compared to free motion. In general, by increasing the spring coefficient by 1.6 times, a 40% reduction in displacement has been calculated for the free movement mode and a 36% reduction in displacement for the two fixed ends of a polymer or restricted mode. In the case of electro-osmotic flow, in restricted motion, flow is very uniform and smooth and will moves the polymer chain smoothly and is probably a proper option for transferring the bio materials with less dispersion.

In general, in the restricted motion mode, less displacement is reported by increasing the stiffness coefficient, and a decrease of 40% in displacement is achieved by increasing the stiffness coefficient. The pressure gradient method is more suitable if just more displacement is considered.

### Effect of different number of polymer beads on displacement

One of the effective parameters in displacement of the polymer chain, the stretch or compression of a polymer chain is the number of beads. In all the previous test cases, the results were presented with 50 beads, and in this section, the number of beads are changed to 25 and also 100 beads which the variation of displacement in the free and restricted motion modes and dispersion are studied for both external forces.

**Figure 10:**
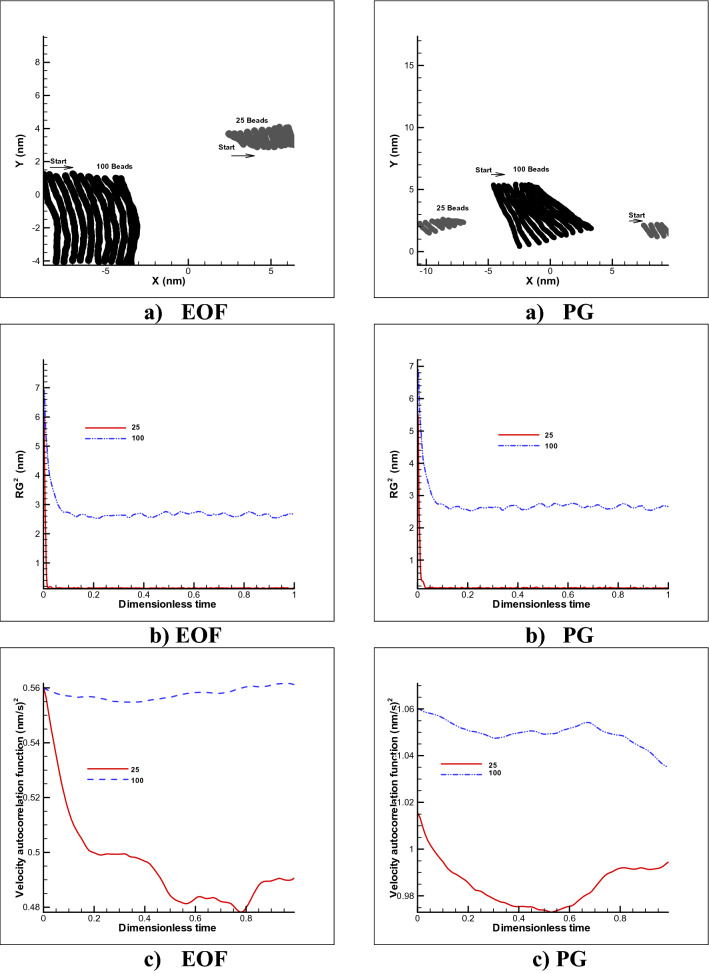
Free movement of a polymer chain in a simple channel (**a**), changes in gyration radius (**b**) and velocity correlation function (**c**) over time considering different n = 25 and n = 100 beads for both pressure gradient and electroosmotic flow test cases.

As shown in Fig. 10, in the free motion mode with PG and spring coefficient of 8000, the polymer chain has tendency to rotates in both 25 and 100 beads, which is expectable due to the nature of the pressure gradient flow, while in the electroosmotic flow due to the uniform velocity profile, the polymer chain keep its state with minimum amount of rotational behavior. There are also insignificant changes to the radius of gyration in the two modes, indicating a slight change in the coil form of the polymer chain, while the motion dynamics or velocity autocorrelation is reported weaker and slower for the EOF mode to PG (almost 50%). It should be reported that for both cases, the radius of gyration (small size of random coil shape) and velocity auto correlation function (dynamics of movement) for 25 beads has lower numerical value to 100 beads.

**Figure 11:**
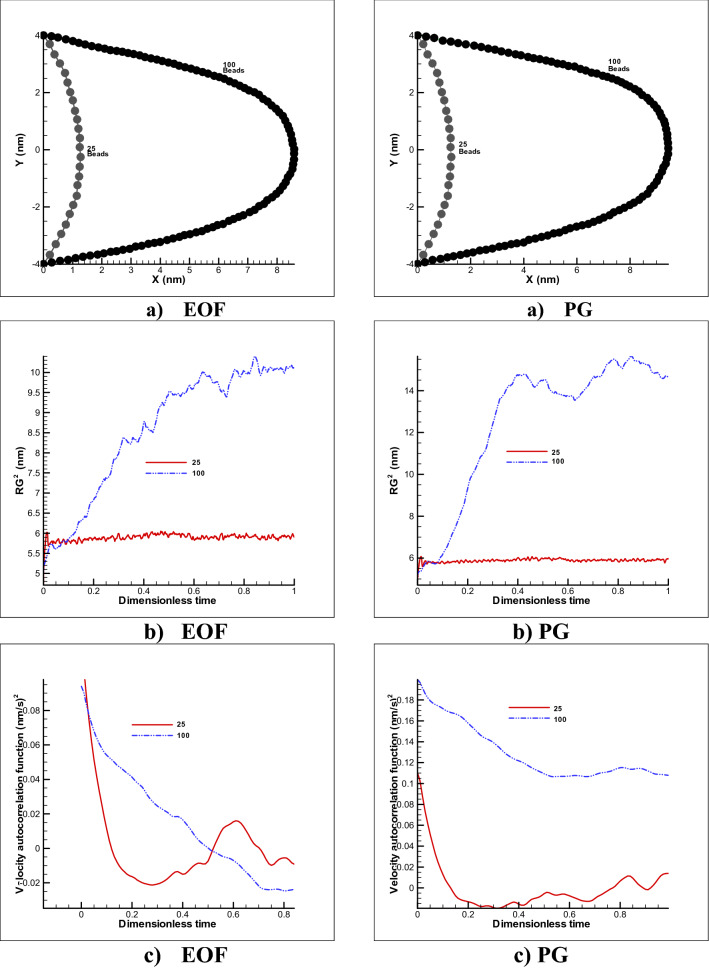
Movement with fixed two ends of a polymer chain in a simple channel (a), changes in gyration radius (**b**) and velocity correlation function (**c**) over time considering different n = 25 and n = 100 beads for both pressure gradient and electroosmotic flow test cases.

From another aspect, restricted motion mode in Fig. 11 shows a conspicuous displacement for 100 beads compared to 25 beads which more beads and springs can naturally stretch easily. Also, EOF mode gives slighter displacement compared to PG (9%). In case of 100 beads, the size of polymer chain will change due to increasing the radius of gyration over time for both cases (unlike PG) while in the case of 25 beads, small variation or final formation of polymer chain will be resulted so fast.

## Conclusion

In this study, the behavior of a polymer chain in a simple channel was simulated under different external forces (GP, EOF), considering two different condition including the free and fixed two ends for a polymer using dissipation particle dynamics (DPD) method.

Initially, in order to evaluate the simulation numerical code, the results were evaluated in a simple channel under the influence of GP and EOF in a fully developed state, with analytical results and the accuracy of the results were reported with an error of less than 10%. Next, the polymer chain was placed in a simple channel and the behavior of the polymer chain was investigated and analyzed for determined time range at GP and EOF mode for variety of spring coefficient. These results include:

- In the case of free movement-PG, about 40% decreases in displacement were observed by increasing the stiffness coefficient by 1.6 times, and the polymer chain was less inclined to twist around itself and it decreased in oscillations of the radius of gyration, and perturbations in the VACF were observed by passing time.
- In the case EOF, dependency to spring coefficient was less than PG and almost constant displacement was reported for mentioned condition. EOF provides proper transition without dispersion.
- In the case of the fixed two ends, with increasing spring coefficient, the tendency to stretch decreases and with increasing 1.6 times the spring stiffness coefficient, about 36% decrease in polymer chain at PG was obtained and in the same behavior, for EOF 39% decline in stretching was formed. Decreases in gyration radius and turbulence in the velocity correlation function have also been observed for both cases by increasing the spring coefficients.
- Also, increasing the number of the polymer beads (also springs) gives higher radius of gyration and VACF. In the case of EOF provides a slight less dispersion displacement for both cases.

It can be concluded that the EOF results in less dispersion for transition of free polymer chain and the same displacement for fixed two ends using DPD method which this method is a powerful method in simulating the motion of micro/nanofluid along with the polymer chain and investigating the complex behavior of the fluid polymer under different condition.

## Data Availability

The data that support the findings of this study are available from the corresponding author upon reasonable request.
